# Additive value of dobutamine stress echocardiography in patients with an anomalous origin of a coronary artery

**DOI:** 10.1007/s12471-014-0648-3

**Published:** 2015-01-27

**Authors:** Heleen Lameijer, Jozine M. ter Maaten, Robbert C. Steggerda

**Affiliations:** 1 Department of Cardiology, Martini Hospital, van Swietenplein 1, 9728 NT Groningen, the Netherlands; 2 Department of Emergency Medicine, University Medical Centre Groningen, Groningen, the Netherlands

## Abstract

An anomalous origin of a coronary artery (AOCA) is the second most common cause of non-traumatic sudden cardiac death in young athletes. Patients with a malignant course of an AOCA of the right coronary artery only need surgical correction when myocardial ischaemia is detected. An AOCA and its malignant or benign course can be detected by coronary angiography, coronary computed tomography or cardiac magnetic resonance imaging. Detection of ischaemia can be more difficult since even a negative maximal-effort stress ECG does not exclude a potential lethal coronary anomaly. Also, there are no case series or trials showing sensitivity or specificity for any form of ischaemia detection for AOCA in the literature. Although not described previously in adults, dobutamine stress echocardiography was previously described in a paediatric population with AOCA. We are the first to describe ischaemia detection by dobutamine stress echocardiography in three adult patients with an AOCA of the right coronary artery who were subsequently referred for surgery.

**Electronic supplementary material:**

The online version of this article (doi: 10.1007/s12471-014-0648-3) contains supplementary material, which is available to authorized users.

## Introduction

**Fig. 1:**
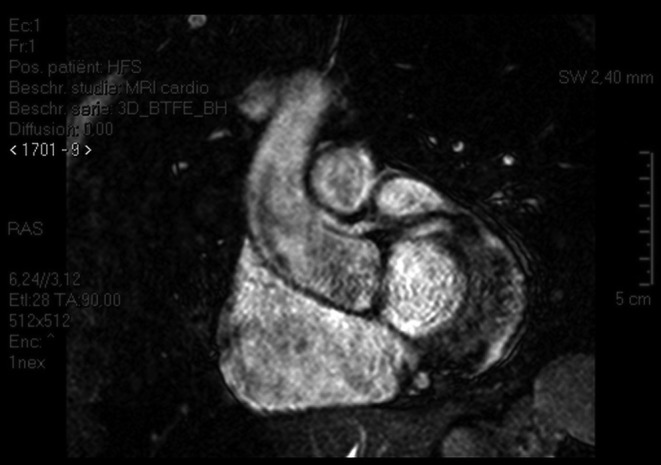
Coronary angiography of a patient showing an aberrant *left* coronary artery originating from the *right* coronary ostium

**Fig. 2:**
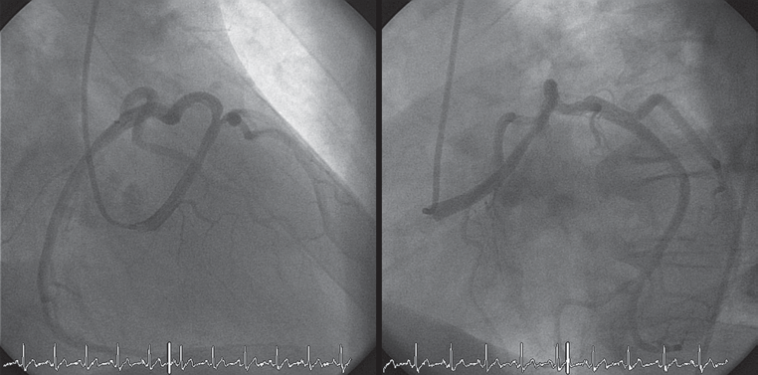
Coronary computed tomography scan of a patient showing an aberrant origin of the *right* coronary artery with a malignant inter-arterial course

An anomalous origin of a coronary artery (AOCA) is the second most common cause of non-traumatic sudden cardiac death in young athletes [1]. Patients diagnosed with a malignant, inter-arterial course of an AOCA of the left coronary artery (Fig. 1) are usually referred for cardiac surgery according to the guidelines. Patients with a malignant course of an AOCA of the right coronary artery (Fig. 2) only need surgical correction when myocardial ischaemia is detected. In three patients with an AOCA of the right coronary artery we detected ischaemia using dobutamine stress echocardiography. For more detailed case descriptions and discussion, see our online supplementary material.

## Discussion

An AOCA and its malignant or benign course can be detected by coronary angiography (best including a right oblique view), coronary computed tomography or cardiac magnetic resonance imaging [2]. Detection of ischaemia can be more difficult since even a negative maximal-effort stress ECG does not exclude a potential lethal coronary anomaly [1, 3, 4]. Also there are no case series or trials showing sensitivity or specificity for any form of ischaemia detection for AOCA in the literature. Though not described previously in adults, dobutamine stress echocardiography was previously described in a paediatric population with AOCA [5]. We are the first to describe ischaemia detection by dobutamine stress echocardiography in three adult patients with an AOCA of the right coronary artery who were referred subsequently for surgery.

## Conclusion

Since normal routine exercise electrocardiography test does not exclude ischaemia in patients with AOCA, we suggest the use of dobutamine stress echocardiography in patients with AOCA.

## Electronic supplementary material

(DOCX 1493 kb)
