# Supplementary material for: Additive value of dobutamine stress echocardiography in patients with an anomalous origin of a coronary artery
Source: Neth Heart J. 2015 Jan 27;23(2):139–40. doi: 10.1007/s12471-014-0648-3 (PMC4315785; doi:10.1007/s12471-014-0648-3)
Supplement: Supplementary file 1 — (DOCX 1493 kb) [file 12471_2014_648_MOESM1_ESM.docx]

**Online supplemental material: extended case description and comment from authors**

**Additive value of dobutamine stress echocardiography in three patients with an anomalous origin of a coronary artery**

Heleen Lameijer^1,2^, MD, Jozine M. ter Maaten^1^, MD, Robbert C. Steggerda, MD^1^

^1^ Department of Cardiology, Martini Hospital, Groningen, the Netherlands.

^2^ Department of Emergency Medicine, University Medical Centre Groningen, Groningen, the Netherlands

**Introduction**

Anomalous origin of a coronary artery (AOCA) is uncommon in the general population. AOCA has, however, been associated with sudden cardiac death (SCD). In fact, it is considered responsible for 17% of all SCD in a young athlete population [1]. These anomalies are rarely identified during life and may be first recognised at autopsy, due to either the absence of specific symptoms or the lack of sensitivity of routine exercise testing [1-3]. We describe the additive value of dobutamine stress echocardiography in three patients with an AOCA for detection of ischaemia and its role in further management decisions.

**Case 1.** Our first patient is a 56-year-old Afro-American man, who presented with chest pain during stress and exercise. Electrocardiography exercise stress testing was normal. Due to persistent exercise-induced chest pain, a coronary angiogram (CAG) was performed. This revealed a right coronary artery originating from the left coronary sinus of Valsalva (ARCA). A malignant inter-arterial course between the aorta and pulmonary artery was confirmed by cardiac magnetic resonance imaging (CMR) (Fig. 1). Additional dobutamine stress echocardiography revealed ischaemia of the right ventricle. The patient was treated with a surgical unroofing procedure of the right coronary artery [4].

**Case 2.** Our second patient, a 54-year-old Caucasian man with a history of atrial fibrillation and aortic valve regurgitation, presented with typical progressive exercise-induced chest pain. Echocardiography showed a bicuspid aortic valve with combined moderate stenosis and moderate regurgitation. A CAG was performed and showed an aberrant left coronary artery (ALCA) originating from the right coronary ostium (Fig. 2). Coronary computed tomography (CCT) scan confirmed an ALCA with a malignant inter-arterial course. Dobutamine stress echocardiography revealed ischaemia. The patient underwent surgery with a Bentall procedure and bypass graft of the left internal mammary artery on the left anterior descending coronary artery.

**Case 3.** Our third patient, a 43-year-old Caucasian man with a history of paroxysmal atrial fibrillation, presented with chest pain and dyspnoea. A CCT scan revealed an ARCA with a malignant inter-arterial course which was also visible on the CAG (Fig. 3). Exercise stress testing did elicit chest pain but without significant ST changes. A dobutamine stress echocardiogram revealed ischaemia of the inferior wall. The patient was therefore treated with an unroofing procedure of the right coronary artery.

**Discussion**

**Prevalence, types** **and consequences**

The prevalence of coronary anomalies has been estimated from 0.3% to 1.33% [5-7]. AOCA is one of these anomalies, and comes in different forms. Among these are the anomalous origin of the right (ARCA, prevalence 0.11-0.16% [6,8]) or left (ALCA, prevalence 0.02-0.04%[6,8]) coronary artery from the opposite sinus of Valsalva. They may run either anterior to the pulmonary artery, inter-arterial (between aorta and pulmonary artery), posterior to the aorta or intra-septally. Of these variations, only ‘the malignant inter-arterial course’ is considered a risk for SCD. In addition, ALCA has more often been associated with SCD than ARCA [9-11]. Surgery is therefore recommended in patients with ALCA, while for ARCA surgery is only recommended when ischaemia is detected.

**Detection**

Symptoms of syncope and chest pain seem to occur mostly in patients with ALCA, whereas ARCA is often clinically silent (63%) [12,13]. Congenital coronary abnormalities and their malignant or benign course can be detected by CAG (best including a right oblique view), CCT or CMR [14]. Detection of ischaemia can be difficult since even a negative maximal-effort stress ECG does not exclude a potentially lethal coronary anomaly [1-3,12]. Also, there are no large case series or trials showing sensitivity or specificity for any form of ischaemia detection for AOCA in the literature. Although not described previously in adults, dobutamine stress echocardiography was previously described for evaluation of myocardial perfusion in a paediatric population with AOCA [15]. To our knowledge, we were the first to describe detection of ischaemia due to AOCA in three adults using dobutamine stress echocardiography.

**Management**

In patients diagnosed with ALCA with an inter-arterial course, surgical intervention is usually indicated according to the guidelines. However, when a malignant course is found by chance in patients with an advanced age, its clinical significance may be questionable. In patients with an inter-arterial course of an ARCA, surgical intervention is only indicated when ischaemia has been detected. By using dobutamine stress echocardiography, ischaemia was detected in two patients with an ALCA and ARCA, resulting in referral for surgery. Dobutamine stress echocardiography may thus be helpful especially in patients with ARCA, but also in patients with ALCA when clinical significance is doubted.

**Conclusions**

AOCA is uncommon but possibly lethal. Patients with a malignant inter-arterial course of an ALCA or ARCA with proven ischaemia may be at risk for SCD and may therefore gain from surgical intervention. Since a normal routine exercise electrocardiography test does not exclude ischaemia, we suggest the use of dobutamine stress echocardiography in patients with AOCA.

**References**

[1] Maron BJ, Doerer JJ, Haas TS, Tierney DM, Mueller FO. Sudden deaths in young competitive athletes: analysis of 1866 deaths in the United States, 1980-2006. Circulation 2009; 119:1085-1092.

[2] Cohen M, Berger S. The electrocardiogram as an adjunct in diagnosing congenital coronary arterial anomalies. Cardiol Young 2010; 20 Suppl 3:59-67.

[3] Chu E, Cheitlin MD. Diagnostic considerations in patients with suspected coronary artery anomalies. Am Heart J 1993; 126:1427-1438.

[4] Romp RL, Herlong JR, Landolfo CK*, et al.* Outcome of unroofing procedure for repair of anomalous aortic origin of left or right coronary artery. Ann Thorac Surg 2003; 76:589-95; discussion 595-6.

[5] Click RL, Holmes DR,Jr, Vlietstra RE, Kosinski AS, Kronmal RA. Anomalous coronary arteries: location, degree of atherosclerosis and effect on survival--a report from the Coronary Artery Surgery Study. J Am Coll Cardiol 1989; 13:531-537.

[6] Yamanaka O, Hobbs RE. Coronary artery anomalies in 126,595 patients undergoing coronary arteriography. Cathet Cardiovasc Diagn 1990; 21:28-40.

[7] Alexander RW, Griffith GC. Anomalies of the coronary arteries and their clinical significance. Circulation 1956; 14:800-805.

[8] Lytrivi ID, Wong AH, Ko HH*, et al.* Echocardiographic diagnosis of clinically silent congenital coronary artery anomalies. Int J Cardiol 2008; 126:386-393.

[9] Eckart RE, Scoville SL, Campbell CL*, et al.* Sudden death in young adults: a 25-year review of autopsies in military recruits. Ann Intern Med 2004; 141:829-834.

[10] Frescura C, Basso C, Thiene G*, et al.* Anomalous origin of coronary arteries and risk of sudden death: a study based on an autopsy population of congenital heart disease. Hum Pathol 1998; 29:689-695.

[11] Cheitlin MD, De Castro CM, McAllister HA. Sudden death as a complication of anomalous left coronary origin from the anterior sinus of Valsalva, A not-so-minor congenital anomaly. Circulation 1974; 50:780-787.

[12] Basso C, Maron BJ, Corrado D, Thiene G. Clinical profile of congenital coronary artery anomalies with origin from the wrong aortic sinus leading to sudden death in young competitive athletes. J Am Coll Cardiol 2000; 35:1493-1501.

[13] Roberts WC, Siegel RJ, Zipes DP. Origin of the right coronary artery from the left sinus of valsalva and its functional consequences: analysis of 10 necropsy patients. Am J Cardiol 1982; 49:863-868.

[14] Taylor AM, Thorne SA, Rubens MB*, et al.* Coronary artery imaging in grown up congenital heart disease: complementary role of magnetic resonance and x-ray coronary angiography. Circulation 2000; 101:1670-1678.

[15] Kimball TR. Pediatric stress echocardiography. Pediatr Cardiol 2002; 23:347-357.

**Figures**


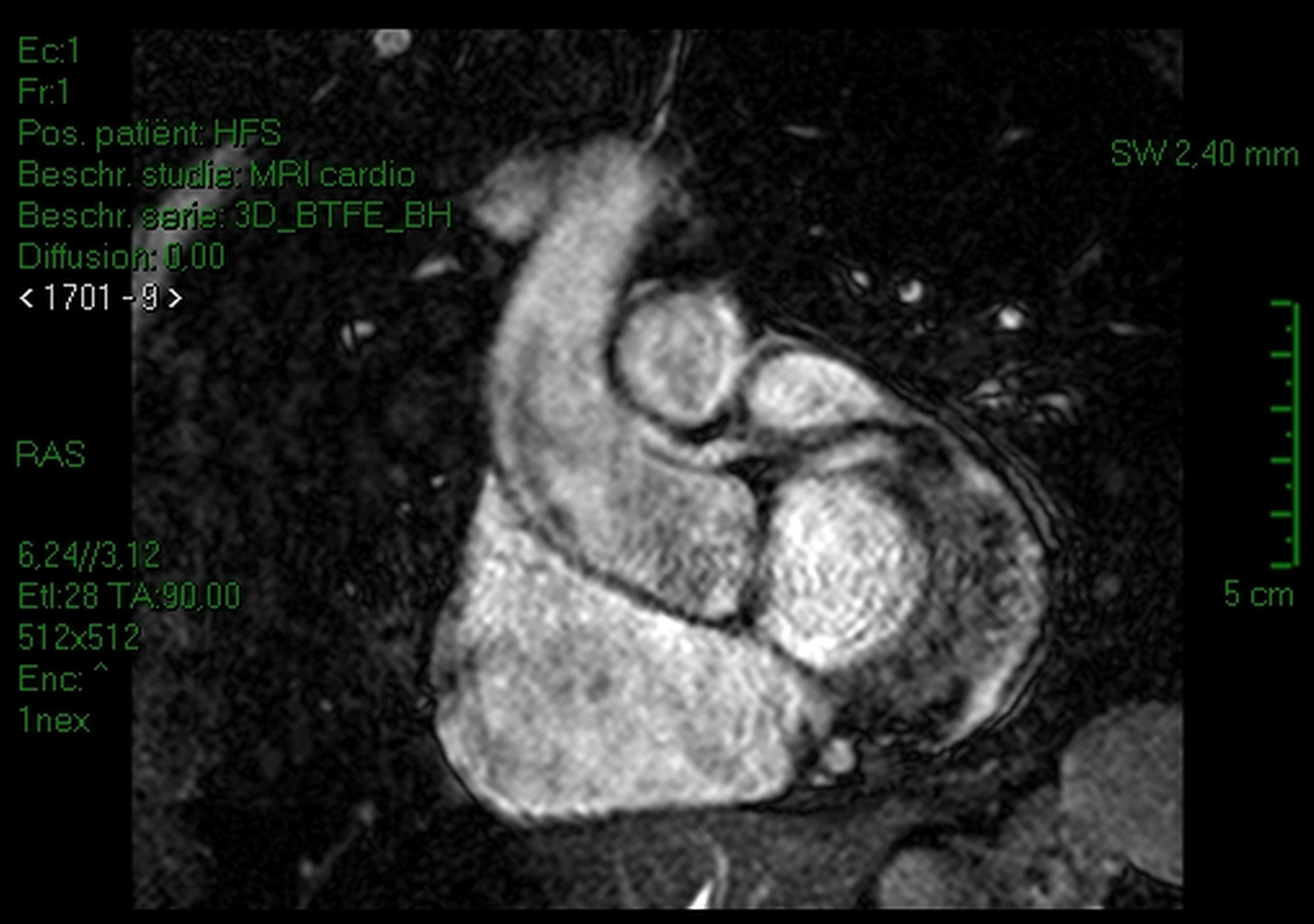


**Fig. 1** Magnetic resonance imaging with perfusion of our first patient showing inter-arterial course between the aorta and pulmonary artery.


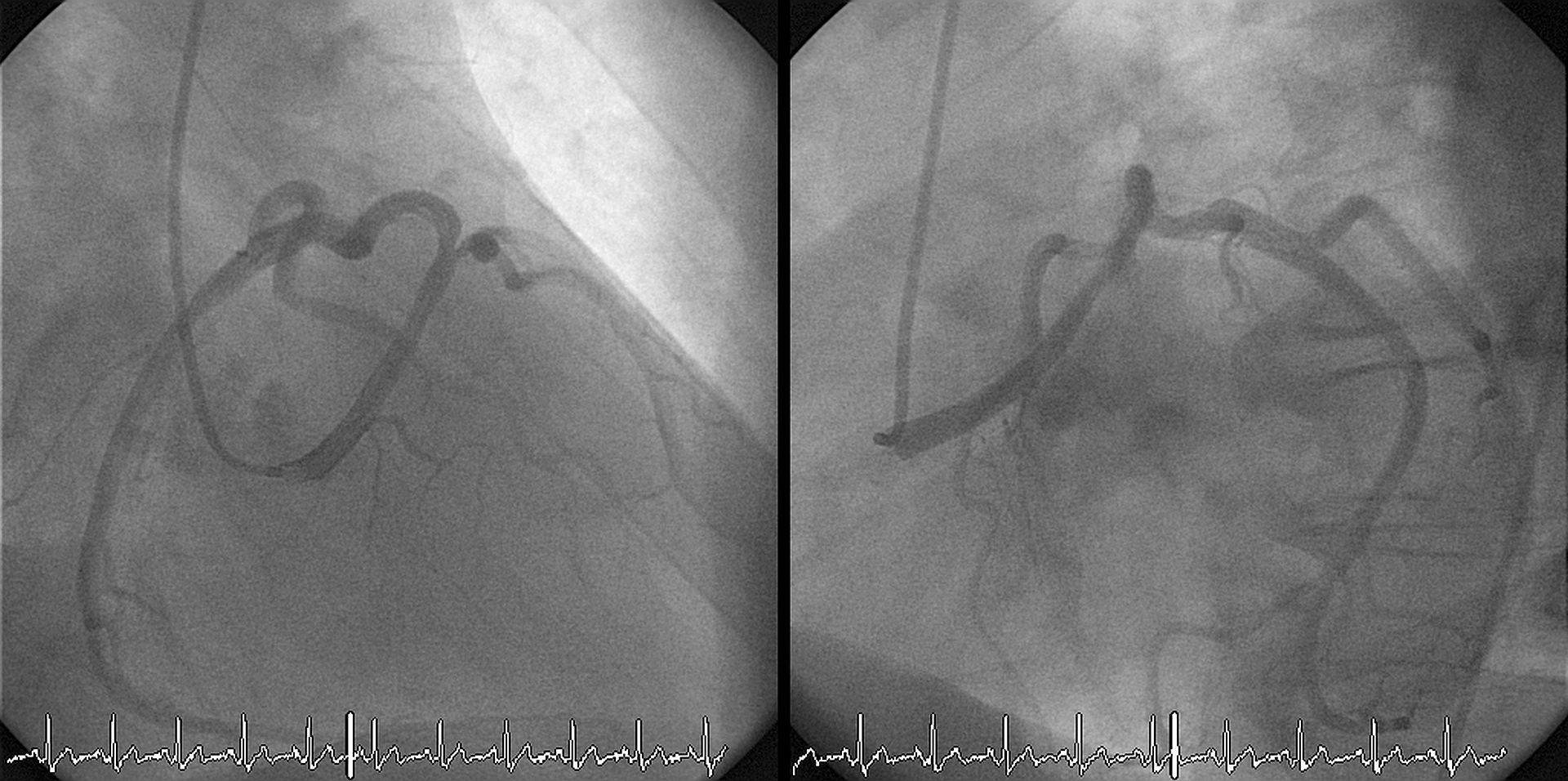


**Fig. 2** Coronary angiography of our second patient showing an aberrant left coronary artery originating from the right coronary ostium.


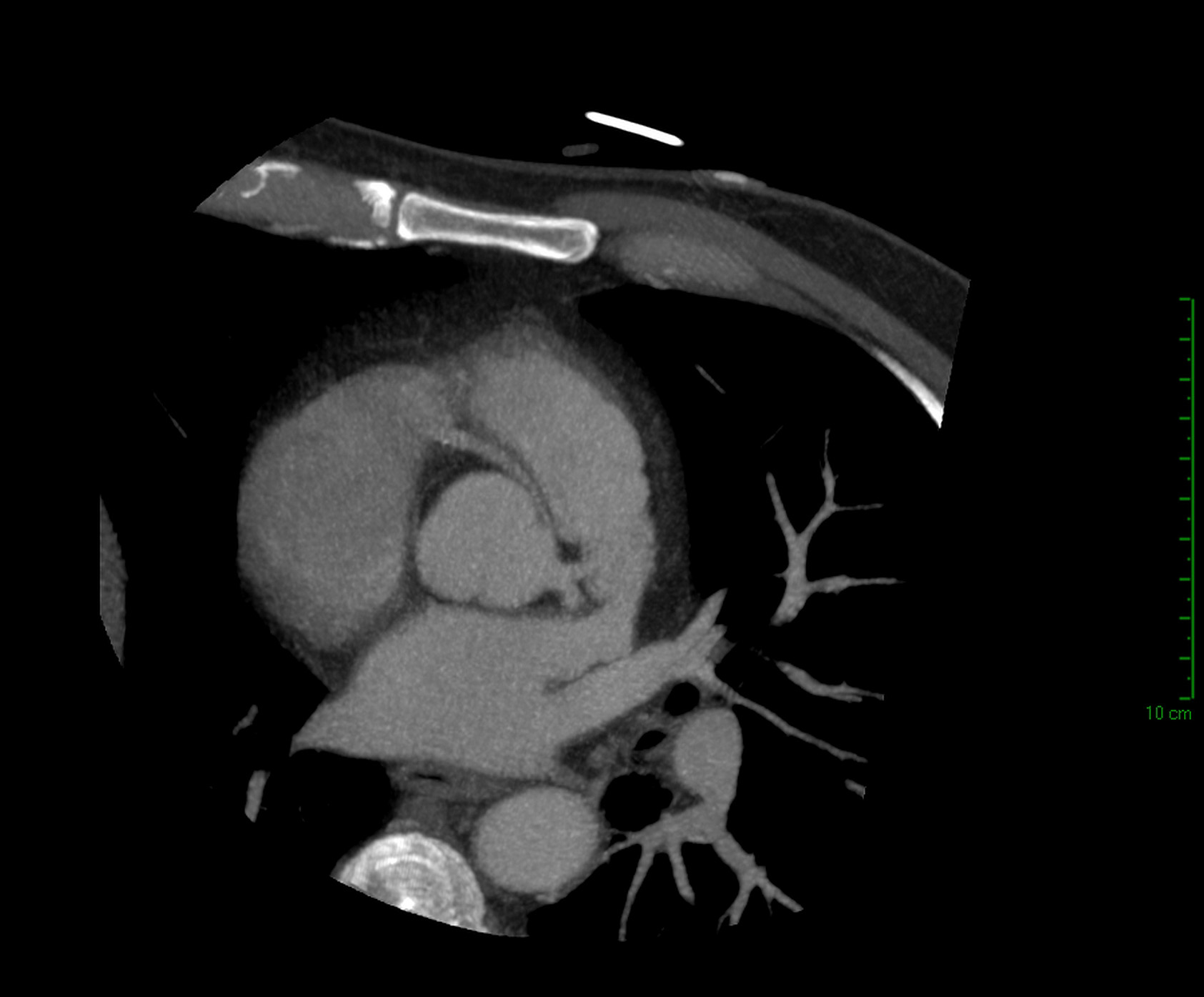


**Fig. 3.** Coronary computed tomography scan of our third patient showing an aberrant origin of the right coronary artery with a malignant inter-arterial course.
